# P016: Device associated infections in adult intensive care units in public versus private hospitals in Egypt

**DOI:** 10.1186/2047-2994-2-S1-P16

**Published:** 2013-06-20

**Authors:** M Abdelfattah, A Elkholy, M Enany, I Beheiry, D Saleh

**Affiliations:** 1 Clinical Pathology, Faculty of Medicine, Cairo University, Cairo, Egypt; 2 Public Health, Faculty of Medicine, Cairo University, Cairo, Egypt

## Introduction

The healthcare system depends on public hospitals including university hospitals and private hospitals in Egypt. Private hospitals have more resources, and healthcare workers work under strict supervision, monitoring and feed- back.

## Objectives

We aimed to compare the DAI rates, patient risk factors and difference in resources between 2 adult medical ICUS, one from a public and one from a private hospital.

## Methods

The study was done as a prospective incidence- based surveillance on 971 adult patients admitted to one medical ICU and one respiratory ICU of Cairo University hospital over a period of 9 months. The same surveillance method was conducted on 250 adult patients in an adult medical ICU from a tertiary- care private hospital. The standardized CDC/NHSN protocols and definitions were used.

## Results

The CLABSI, VAP and CAUTI rates were 10.0, 5.6, 3.2/1000 device days respectively in the medical ICU and 3.5, 28.5, 12.4/1000 device days in the respiratory ICU of the public hospital (CUH) versus 2.3, 0, 1.7 /1000 device days respectively in the private hospital (DAF) ICU (p value= 0.08, < 0.001 and 0.007).

The Central line, ventilator, urinary catheter utilization ratios were 0.19, 0.29, 0.41in the medical ICU and 0.26, 0.27, 0.33 in the respiratory ICU respectively in the CUH, versus 0.57, 0.15, 0.38 respectively in DAF ICU.

The significant risk factor for DAI was APACH score>15% for DAI in both ICUs while the length of hospital stay was a significant risk for DAI CUH ICU only. In CUH ICU, nurse to patient ratio was 1:3 versus 1:1in DAF ICU. Hand hygiene compliance was 50% in CUH versus and 85% in DAF.

## Conclusion

The increase in LOS, decrease in hand hygiene compliance and nurse to patient ratio could explain the higher incidence of DAI in CUH over the private ICUs in Egypt. Rectification of these factors can reduce the rates of DAI in public hospitals.

## Disclosure of interest

None declared

